# Current News

**Published:** 1902-10

**Authors:** 


### Current News.

### AMERICAN SOCIETY OF ORTHODONTISTS.

The second annual meeting of the American Society of Orthodontists, will be held at the Continental Hotel, Philadelphia, October 8, 9, and 10, 1902.

The following papers will be presented:

President's Address.

Normal and Pathological Anatomy of the Alveolar Process and Adjacent Tissue, M. H. Cryer.

Subject to be announced, Edward C. Kirk.

Art in Relation to Orthodontia, Edward H. Angle.

The Deformities of the Superior Maxilla from the Stand-Point of the Rhinologist, C. H. Kohler.

The Causes of Malocclusion, Wm. J. Brady.

Retrusion of Both Jaws with a Single Appliance, R. Ottolengui.

Nasal Occlusion and Septal Deviation in their Relation to Antral Development and Facial Expression, Royal S. Copeland.

Orthodontia from the Stand-Point of a Student, Anna Hopkins.

Distal Movement of Molars and Bicuspids limiting Extraction, Lloyd S. Lourie.

Stationary and Removable Appliances, Alone and in Combination, H. A. Pullen.

Subjects to be announced, W. Booth Pearsall, J. Humphries, J. E. Grevers.

Time will be reserved for the consideration of specimens, pertaining to orthodontia, which any one may care to present. A cordial invitation is extended to the profession.

MILTON T. WATSON,

*Secretary.*

270 WOODWARD AVENUE, DETROIT.

---

### NEW JERSEY STATE BOARD OF DENTAL EXAMINERS.

THE New Jersey State Board of Dental Examiners will hold their fall examinations on Tuesday, October 21, Wednesday, 22, and Thursday, 23, at 9.30 A.M.

Further information may be had of

J. ALLEN OSMUN,
*Secretary.*

588 BROAD STREET, NEWARK, N. J.

---

### NORTHEASTERN DENTAL ASSOCIATION.

THE eighth annual meeting of the Northeastern Dental Association will convene in the City of Worcester, Mass., October 15, 1902, and continue through October 17. This meeting promises to be better than its predecessors in essays, clinics, and exhibits. Invitation is extended to New England dentists, members of their respective State Dental Societies, to attend and join the Association. Remember the dates. One and one-third fares, certificate plan, on all railroads.

EDGAR O. KINSMAN,

*Secretary.*

### TENNESSEE DENTAL ASSOCIATION.

The thirty-fifth annual meeting of the Tennessee Dental Association was held at Mount Eagle, Tenn., July 8, 9, and 10, Dr. J. T. Meadors, presiding.

Thoughtful papers, heated discussions, interesting clinics, a pleasant place of meeting, and a good attendance made this one of the most interesting meetings held for several years. Six new members were added to the roll, and several absentees sent in their dues, thus showing an increased interest in association work.

A committee was appointed and a fund appropriated to cooperate with other societies in the matter of securing aid from the Carnegie Institute at Washington, D. C., to assist in scientific research along certain lines of special interest to the profession.

The following officers were chosen for the ensuing year:

President, Dr. W. K. Slater, Knoxville, Tenn.; First Vice-President, Dr. R. Boyd Bogle, Nashville, Tenn.; Second Vice-President, Dr. W. P. Menzies, Dyersburg, Tenn.; Recording Secretary, Dr. A. S. Page, Columbia, Tenn.; Corresponding Secretary, Dr. J. T. Meadors, Columbia, Tenn.; Treasurer, Dr. J. D. Towner, Pulaski, Tenn.

*Executive Committee.*—Dr. J. W. Peete, Memphis, Tenn., Chairman; Dr. A. R. Melendy, Knoxville, Tenn.; Dr. Jno. R. Beach, Clarksville, Tenn.

Chattanooga, Tenn., was chosen as the next place of meeting, and the date left with the Executive Committee.

A. S. Page,
*Secretary.*

---

### UNION MEETING OF THE SEVENTH AND EIGHTH DISTRICT DENTAL SOCIETIES OF NEW YORK.

There will be a union meeting of the Seventh and Eighth District Dental Societies of New York in Buffalo, October 28 and 29, 1902.

The Business Committee is sparing no effort to make this meeting a success.

B. W. Whipple,
*Recording Secretary.*

